# Supplementary material for: Sex differences in long-term survival after intensive care unit treatment for sepsis: A cohort study
Source: PLoS One. 2023 Feb 24;18(2):e0281939. doi: 10.1371/journal.pone.0281939 (PMC9955961; doi:10.1371/journal.pone.0281939)
Supplement: S1 File — (DOCX) [file pone.0281939.s001.docx]

**Supporting Information**

**Sex differences in long-term survival after intensive care unit treatment for sepsis: a cohort study.**

Kelly Thompson, Naomi Hammond, Michael Bailey, Jai Darvall, Gary Low, Steven McGloughlin, Lucy Modra, David Pilcher.

Contents

[S1 Table. Acute Physiology and Chronic Health Evaluation III Diagnosis Codes used to identify sepsis 3](#_Toc125801609)

[S2 Table. Baseline characteristics of all ICU admissions with sepsis 6](#_Toc125801610)

[S3 Table. Baseline characteristics of the sepsis admissions included compared to those unable to be matched to death index 9](#_Toc125801611)

[S1 Fig. Sepsis survival at 3 years in women and men overall and by age group 10](#_Toc125801612)

[S2 Fig. Sepsis survival at 3 years among hospital survivors in women and men overall and by age group 11](file:///H:\Academic\PROJECTS\ANZICS%20CORE\Paper\AJRCCM\CCF\Journal%20of%20Infection\Supplemental%20Online%20Content_27Jan23.docx#_Toc125801613)

[S4 Table. Impact of individual variables on overall survival (Cox regression models for women, men and comparing men to women). 12](#_Toc125801614)

[S3 Fig. COVID patients only. 16](#_Toc125801615)

[S4 Fig. COVID patients excluded. 16](#_Toc125801616)

| S1 Table. Acute Physiology and Chronic Health Evaluation III Diagnosis Codes used to identify sepsis | | | | | | | | |
| --- | --- | --- | --- | --- | --- | --- | --- | --- |
| ***APACHE III Code*** | ***Code name*** | ***Gastro-intestinal (GI)*** | ***Neurological*** | ***Other/ unknown*** | ***Renal/ urinary*** | ***Respiratory*** | ***Skin &***  ***soft tissue*** | ***Total*** |
| ***Non-operative*** |  |  |  |  |  |  |  |  |
| 109 | Other cardiovascular disease | 0 | 0 | 344 | 0 | 0 | 0 | 344 |
| 210 | Parasitic pneumonia | 0 | 0 | 0 | 0 | 212 | 0 | 212 |
| 212 | Bacterial pneumonia | 0 | 0 | 0 | 0 | 8,875 | 0 | 8,875 |
| 213 | Viral pneumonia | 0 | 0 | 0 | 0 | 2,593 | 0 | 2,593 |
| 313 | Other GI inflammatory disease | 753 | 0 | 0 | 0 | 0 | 0 | 753 |
| 404 | Neurologic infection | 0 | 1,144 | 0 | 0 | 0 | 0 | 1,144 |
| 501 | Sepsis, other than urinary | 1,654 | 0 | 6,949 | 147 | 2,298 | 1,563 | 12,611 |
| 502 | Sepsis, of urinary tract origin | 0 | 0 | 0 | 4,063 | 0 | 0 | 4,063 |
| 503 | Sepsis with shock, other than urinary | 0 | 0 | 13,728 | 0 | 0 | 0 | 13,728 |
| 504 | Sepsis of urinary tract origin with shock | 0 | 0 | 0 | 3,819 | 0 | 0 | 3,819 |
| 901 | Renal disorders | 0 | 0 | 0 | 151 | 0 | 0 | 151 |
| 1102 | Cellulitis/soft tissue infection | 0 | 0 | 0 | 0 | 0 | 625 | 625 |
| ***Post-operative*** |  |  |  |  |  |  |  |  |
| 1301 | Respiratory infection | 0 | 0 | 0 | 0 | 630 | 0 | 630 |
| 1406 | Cholecystitis/Cholangitis | 3,253 | 0 | 0 | 0 | 0 | 0 | 3,253 |
| 1409 | Fistula/Abscess surgery | 579 | 0 | 0 | 0 | 0 | 0 | 579 |
| 1412 | Peritonitis | 578 | 0 | 0 | 0 | 0 | 0 | 578 |
| 1506 | Other neurologic disease | 0 | 145 | 0 | 0 | 0 | 0 | 145 |
| 1904 | Cellulitis/soft tissue infection | 0 | 0 | 0 | 0 | 0 | 1,613 | 1,613 |
| ***Diagnostic subcodes (some patients only have the main diagnosis listed, without a subcode)*** | | | | | | | | |
| ***APACHE III*** | ***Subcode name*** | ***Freq.*** | ***Percent*** | ***Cum.*** |  |  |  |  |
| 109.06 | Endocarditis | 344 | 0·62 | 0·62 |  |  |  |  |
| 210.01 | Pneumonia fungal | 76 | 0·14 | 0·75 |  |  |  |  |
| 210.02 | Atelactasis | 93 | 0·17 | 0·92 |  |  |  |  |
| 210.03 | Suspected or confirmed pandemic infection | 13 | 0·02 | 0·94 |  |  |  |  |
| 212.01 | Pneumonia, bacterial | 5,663 | 10·16 | 11·11 |  |  |  |  |
| 212.02 | Pneumonia, other | 1,510 | 2·71 | 13·82 |  |  |  |  |
| 212.03 | Suspected or confirmed pandemic infection | 216 | 0·39 | 14·21 |  |  |  |  |
| 213.01 | Pneumonia, viral | 1,610 | 2·89 | 17·1 |  |  |  |  |
| 213.02 | Suspected or confirmed pandemic infection | 487 | 0·87 | 17·97 |  |  |  |  |
| 311.01 | Pancreatitis | 1 | 0 | 17·97 |  |  |  |  |
| 313.01 | Cholangitis | 558 | 1 | 18·97 |  |  |  |  |
| 313.03 | GI Abscess/cyst | 86 | 0·15 | 19·13 |  |  |  |  |
| 313.05 | Peritonitis | 109 | 0·2 | 19·32 |  |  |  |  |
| 404.01 | Abscess, neurologic | 107 | 0·19 | 19·52 |  |  |  |  |
| 404.02 | Encephalitis | 482 | 0·87 | 20·38 |  |  |  |  |
| 404.03 | Meningitis | 398 | 0·71 | 21·09 |  |  |  |  |
| 404.04 | Suspected or confirmed pandemic infection | 12 | 0·02 | 21·12 |  |  |  |  |
| 501.01 | Sepsis, cutaneous/soft tissue | 1,563 | 2·81 | 23·92 |  |  |  |  |
| 501.02 | Sepsis, GI | 1,654 | 2·97 | 26·89 |  |  |  |  |
| 501.03 | Sepsis, gynaecological | 147 | 0·26 | 27·15 |  |  |  |  |
| 501.04 | Sepsis, other | 1,972 | 3·54 | 30·69 |  |  |  |  |
| 501.05 | Sepsis, pulmonary | 2,298 | 4·12 | 34·82 |  |  |  |  |
| 501.06 | Sepsis, unknown | 2,882 | 5·17 | 39·99 |  |  |  |  |
| 501.07 | Suspected or confirmed pandemic infection | 180 | 0·32 | 40·31 |  |  |  |  |
| 502.01 | Sepsis, renal/UTI | 3,205 | 5·75 | 46·07 |  |  |  |  |
| 503.01 | Sepsis with shock, not UTI | 10,891 | 19·55 | 65·61 |  |  |  |  |
| 503.02 | Suspected or confirmed pandemic infection | 173 | 0·31 | 65·92 |  |  |  |  |
| 504.01 | Sepsis, with shock urinary tract | 3,114 | 5·59 | 71·51 |  |  |  |  |
| 901.04 | Renal infection/abscess | 151 | 0·27 | 71·78 |  |  |  |  |
| 1102.01 | Arthritis, rheumatoid | 505 | 0·91 | 72·69 |  |  |  |  |
| 1102.02 | Arthritis, septic | 7 | 0·01 | 72·7 |  |  |  |  |
| 1301.01 | Infection/abscess, other surgery for | 630 | 1·13 | 73·83 |  |  |  |  |
| 1406.01 | Cholecystectomy/cholangitis, surgery for gallbladder removal | 2,700 | 4·85 | 78·68 |  |  |  |  |
| 1409.01 | Fistula/abscess, surgery for (not inflammatory bowel disease) | 411 | 0·74 | 79·42 |  |  |  |  |
| 1409.02 | GI abscess/cyst-primary, surgery for  (for complications of GI surgery, see 1408.03) | 168 | 0·3 | 79·72 |  |  |  |  |
| 1412.01 | Peritonitis, for surgery | 480 | 0·86 | 80·58 |  |  |  |  |
| 1506.01 | Abscess/infection cranial, surgery for | 145 | 0·26 | 80·84 |  |  |  |  |
| 1904.01 | Cellulitis and localized soft tissue infections, surgery for | 1,328 | 2·38 | 83·22 |  |  |  |  |
| No subcode listed |  | 9,347 | 16·78 | 100 |  |  |  |  |

| S2 Table. Baseline characteristics of all ICU admissions with sepsis | | | | | | | | |
| --- | --- | --- | --- | --- | --- | --- | --- | --- |
| **Characteristics** | | **Total** | | | **Men** | | **Women** | |
|  |  | **(n = 55,710)** | | | **(n = 31, 467)** | | **(n = 24,243)** | |
| Age, mean (SD) | | 65·0 | | (16·5) | 66·3 | (15·8) | 63·4 | (17·2) |
| **Body mass index (BMI)**, | |  | |  |  |  |  |  |
| mean (SD) | | 29·8 | | (9·1) | 29·4 | (8·3) | 30·4 | (10·1) |
| BMI <18·5, No. (%) | | 1,089 | | (2) | 494 | (1·6) | 595 | (2·5) |
| BMI 18·5 to 24·9, No. (%) | | 7,787 | | (14) | 4,413 | (14) | 3,374 | (13·9) |
| BMI 25 to 29·9, No. (%) | | 8,103 | | (14·5) | 5,101 | (16·2) | 3,002 | (12·4) |
| BMI 30 to 34·9, No. (%) | | 5,016 | | (9) | 2,993 | (9·5) | 2,023 | (8·3) |
| BMI 35+, No. (%) | | 5,725 | | (10·3) | 2,800 | (8·9) | 2,925 | (12·1) |
| BMI missing, No. (%) | | 27,996 | | (50·2) | 15,670 | (49·8) | 12,326 | (50·8) |
| APACHE II score, mean (SD) | | 19·1 | | (7·6) | 19·3 | (7·6) | 18·9 | (7·5) |
| APACHE III score, mean (SD) | | 64·4 | | (24·6) | 65·5 | (24·4) | 63·0 | (24·8) |
| SOFA score day 1, mean (SD) | | 5·4 | | (2·8) | 5·6 | (2·9) | 5·2 | (2·8) |
| ANZROD, mean (SD) | | 14·4 | | (19·1) | 15·0 | (19·4) | 13·6 | (18·81) |
| **Source of sepsis,** No. (%) | |  | |  |  |  |  |  |
| Gastro-intestinal | | 6,817 | | (12·2) | 3,892 | (12·4) | 2,925 | (12·1) |
| Neurological | | 1,289 | | (2·3) | 696 | (2·2) | 593 | (2·4) |
| Other/unknown | | 21,021 | | (37·7) | 12,300 | (39·1) | 8,721 | (36·0) |
| Renal/urinary/gynae | | 8,180 | | (14·7) | 3,919 | (12·5) | 4,261 | (17·6) |
| Respiratory | | 14,608 | | (26·2) | 8,445 | (26·8) | 6,163 | (25·4) |
| Skin & soft-tissue | | 3,801 | | (6·8) | 2,219 | (7·1) | 1,582 | (6·5) |
| **Septic shock,** No. (%) | | 28,694 | | (51·5) | 16,062 | (51·0) | 12,362 | (51·0) |
| Medical admission, No. (%) | | 49,918 | | (89·6) | 27,516 | (87·4) | 21,402 | (88·3) |
| **Pre-existing chronic morbidities,** No. (%) | | | |  |  |  |  |  |
| Respiratory disease | | 8741 | | (15·7) | 3184 | (10·1) | 2557 | (10·5) |
| Cardiac disease | | 5643 | | (10·1) | 3482 | (11·1) | 2161 | (8·9) |
| Liver disease | | 1360 | | (2·4) | 837 | (2·7) | 523 | (2·2) |
| Kidney disease | | 3267 | | (5·9) | 2214 | (7·0) | 1413 | (5·8) |
| Immunosuppressed (therapy) | | 7180 | | (12·9) | 4003 | (12·7) | 3177 | (13·1) |
| Immuno-suppressed (disease) | | 3594 | | (6·4) | 2073 | (6·6) | 1521 | (6·3) |
| History of lymphoma | | 1402 | | (2·52 | 884 | (2·8) | 518 | (2·1) |
| History of metastases | | 3102 | | (5·6) | 1881 | (6·0) | 1221 | (5·0) |
| History of leukaemia | | 2123 | | (3·8) | 1354 | (4·3) | 769 | (3·2) |
| **Clinical Frailty Score** | |  | |  |  |  |  |  |
| CFS, mean (SD) | | 4·0 | | (1·6) | 4·1 | (1·6) | 4·0 | (1·5) |
| Not frail (CFS 1-4), No. (%) | | 24,886 | | (44·6) | 14,247 | (45·3) | 10,639 | (33·8) |
| CFS (5-6), No. (%) | | 10,110 | | (18·1) | 5,453 | (17·3) | 4,657 | (14·8) |
| CFS (7-8), No. (%) | | 2,797 | | (5·0) | 1,475 | (4·7) | 1,322 | (4·2) |
| CFS unknown, No. (%) | | 17,923 | | (32·1) | 10,296 | (32·7) | 7,627 | (24·2) |
| **Clinical characteristics within 24 hours of admission (SD)** | | | | | | | | |
| Highest temperature (˚C), mean (SD) | | 37·55 | | (1·0) | 37·56 | (1·0) | 37·53 | (1·0) |
| Highest heart rate (bpm), mean (SD) | | 106 | | (23) | 106 | (24) | 107 | (23) |
| Lowest mean arterial pressure (mmHg), mean (SD) | | 63 | | (11) | 64 | (11) | 62 | (11) |
| **Laboratory characteristics within 24 hours of admission Median (IQR)** | | | | | | | | |
| Highest white cell count (x10^9^/L), mean (SD) | | 13 | | (8-19) | 13 | (8-19) | 13 | (9-19) |
| Highest lactate (mmol/L), mean (SD) | | 1·8 | | (1-2·8) | 1·8 | (1-2·9) | 1·7 | (1-2·8) |
| Lactate <2 (%), No. (%) | | 13,571 | | (44·2%) | 13,571 | (43·1%) | 11,076 | (45·7%) |
| Lactate 2 to 3·9, No. (%) | | 8,086 | | (24·6%) | 8,086 | (25·7%) | 5,619 | (23·2%) |
| Lactate 4 to 5·9, No. (%) | | 1,938 | | (6%) | 1,938 | (6·2%) | 1,383 | (5·7%) |
| Lactate 6 to 7·9, No. (%) | | 745 | | (2·4%) | 745 | (2·4%) | 572 | (2·4%) |
| Lactate 8 to 9·9, No. (%) | | 401 | | (1·2%) | 401 | (1·3%) | 288 | (1·2%) |
| Lactate 10 to 11·9, No. (%) | | 226 | | (0·7%) | 226 | (0·7%) | 171 | (0·7%) |
| Lactate 12 to 13·9, No. (%) | | 146 | | (0·5%) | 146 | (0·5%) | 119 | (0·5%) |
| Lactate 14+, No. (%) | | 252 | | (0·9%) | 252 | (0·8%) | 231 | (1%) |
| Lactate missing, No. (%) | | 6,102 | | (19·5%) | 6,102 | (19·4%) | 4,784 | (19·7%) |
| Creatinine (µmol/L), mean (SD) | | 111 | | (67-187) | 13,571 | (76-202) | 92 | (58-165) |
| Bilirubin (µmol/L), mean (SD) | | 13 | | (8-24) | 8,086 | (9-26) | 12 | (7-21) |
| **Organ supportive therapies No. (%)** | |  | |  |  |  |  |  |
| Inotropes/vasopressor use | | 28,729 | | (51·6) | 16,349 | (52·0) | 12,380 | (51·1) |
| Invasive mechanical ventilation | | 13,058 | | (23·4) | 7708 | (24·5) | 5353 | (22·1) |
| Renal replacement therapy | | 4232 | | (7·6) | 2582 | (8·2) | 1650 | (6·8) |
| **Source of Hospital admission No. %** | |  | |  |  |  |  |  |
| Home | | 41,343 | | (74·2) | 23,335 | (74·2) | 18,008 | (57·1) |
| Other acute hospital (not ICU) | | 10,847 | | (19·5) | 6,131 | (19·5) | 4,716 | (15·0) |
| Nursing home / chronic care / palliative care | | | 1,036 | (1·8) | 537 | (1·7) | 499 | (1·6) |
| Other hospital ICU | | 1,662 | | (2·9) | 990 | (3·1) | 672 | (2·1) |
| Rehabilitation facility | | 389 | | (0·7) | 218 | (0·7) | 171 | (0·5) |
| Other (incl. mental health, inborn & unknown) | 439 | | | (0·8) | 260 | (0·8) | 179 | (0·6) |
| **Source of ICU admission No. %** | |  | |  |  |  |  |  |
| Operating theatre/recovery | | 6,835 | | (12·2) | 3,980 | (12·6) | 2,855 | (9·1) |
| Emergency department | | 27,113 | | (48·7) | 15,239 | (48·4) | 11,874 | (37·7) |
| Ward | | 15,465 | | (27·7) | 8,726 | (27·7) | 6,739 | (21·4) |
| ICU same hospital | | 44 | | (0·1) | 23 | (0·07) | 21 | (0·06) |
| Other hospital (incl. ICU) | | 6,173 | | (11·1) | 3,450 | (11·0) | 2,723 | (8·7) |
| Other / unknown | | 86 | | (0·1) | 53 | (0·1) | 33 | (0·1) |
|  | |  | |  |  |  |  |  |
| **Medical admission No. %** | | 48918 | | (87·7) | 27516 | (87·4) | 21401 | (88·2) |
| Lowest temperature in first 24 hrs (SD) | | 36·1 | | (0·8) | 36·1 | (0·8) | 36·8 | (0·8) |
| Lowest heart rate in first 24 hrs (SD) | | 73 | | (16) | 72 | (16) | 73 | (16) |
| Highest MAP in first 24 hrs (SD) | | 95·0 | | (17) | 95·0 | (17) | 94 | (17) |
| Lowest WCC in first 24 hrs (SD) | | 12·9 | | (10·4) | 12·8 | (10·7) | 13·0 | (9·8) |
| Urea day 1 (SD) | | 11·7 | | (8·8) | 12·5 | (9·0) | 10·6 | (8·6) |
| pH from ABG (SD) | | 7·4 | | (0·1) | 7·4 | (0·1) | 7·4 | (0·1) |
| paCO2 (SD) | | 38·7 | | (11·7) | 38·5 | (11·7) | 39·0 | (11·7) |
| paO2 (SD) | | 87·6 | | (49·1) | 87·5 | (48·7) | 87·6 | (49·6) |
| FiO2 (SD) | | 0·4 | | (0·2) | 0·4 | (0·2) | 0·4 | (0·2) |
| Died in hospital **No. %** | | 7869 | | (14·1) | 4691 | (14·9) | 3178 | (13·1) |
| Died in ICU **No. %** | | 4791 | | (8·6) | 2834 | (9·0) | 1957 | (8·0) |
| ICU length of stay in days (SD) | | 4·3 | | (6·3) | 4·4 | (6·7) | 4·1 | (5·8) |
| Hospital length of stay in days (SD) | | 16·9 | | (71·7) | 17·2 | (70·5) | 16·5 | (73·2) |
| Readmitted to ICU during the same hospital stay **No. %** | | 2851 | | (5·1) | 1704 | (5·4) | 1147 | (4·7) |
| Duration of invasive mechanical ventilation (hours)  (for those where data is recorded) (SD) | | 128·8 | | (228·0) | 133·0 | (247·1) | 122·4 | (196·4) |
| **Index of Relative Advantage and Disadvantage**** | |  | |  |  |  |  |  |
| Lowest quintile | | 11,559 | | (20·7) | 6,528 | (20·7) | 5,031 | (20·7) |
| Second lowest quintile | | 11,635 | | (20·9) | 6,568 | (20·9) | 5,067 | (20·9) |
| Middle quintile | | 11,191 | | (20·1) | 6,355 | (20·2) | 4,836 | (19·9) |
| Second highest quintile | | 9,845 | | (17·7) | 5,534 | (17·6) | 4,311 | (17·8) |
| Highest quintile | | 11,053 | | (19·8) | 6,218 | (11·2) | 4,835 | (19·9) |
| Unknown (no postcode) | | 433 | | (0·7) | 268 | (0·5) | 165 | (0·7) |

Abbreviations: ICU= intensive care unit, SD = standard deviation, BMI = body mass index, APACHE = Acute Physiology and Chronic Health Evaluation, SOFA = Sequential Organ Failure Assessment, ANZROD = Risk of Death.

| S3 Table. Baseline characteristics of the sepsis admissions included compared to those unable to be matched to death index | | | | | |
| --- | --- | --- | --- | --- | --- |
| **Characteristics** | **Matched** | | **Unmatched** | |  |
|  | **(n = 55,710)** | | **(n = 3024)** | |  |
| Age, mean (SD) | 65·0 | (16·5) | 59·8 | (17·9) |  |
| **Body mass index (BMI)**, |  |  |  |  |  |
| mean (SD) | 29·8 | (9·1) | 29·05 | (9·35) |  |
| APACHE II score, mean (SD) | 19·1 | (7·6) | 17·5 | (7·21) |  |
| APACHE III score, mean (SD) | 64·4 | (24·6) | 59·7 | (21·9) |  |
| SOFA score day 1, mean (SD) | 5·4 | (2·8) | 5·1 | (2·53) |  |
| ANZROD, mean (SD) | 14·4 | (19·1) | 9·79 | (13·26) |  |
| **Source of sepsis,** No. (%) |  |  |  |  |  |
| Gastro-intestinal | 6,817 | (12·2) | 332 | (11) |  |
| Neurological | 1,289 | (2·3) | 90 | (3) |  |
| Other/unknown | 21,021 | (37·7) | 1,057 | (35) |  |
| Renal/urinary/gynae | 8,180 | (14·7) | 439 | (15) |  |
| Respiratory | 14,608 | (26·2) | 891 | (29) |  |
| Skin & soft-tissue | 3,801 | (6·8) | 215 | (7) |  |
| **Septic shock,** No. (%) | 28,694 | (51·5) | 1322 | (43·6) |  |
| Medical admission, No. (%) | 49,918 | (89·6) | 2698 | (89·1) |  |
| **Pre-existing chronic morbidities, No. (%)** | |  |  |  |  |
| Respiratory disease | 8741 | (15·7) | 233 | (7·7) |  |
| Cardiac disease | 5643 | (10·1) | 183 | (6·0) |  |
| Liver disease | 1360 | (2·4) | 75 | (2·4) |  |
| Kidney disease | 3267 | (5·9) | 159 | (5·2) |  |
| Immunosuppressed (therapy) | 7180 | (12·9) | 120 | (3·96) |  |
| Immuno-suppressed (disease) | 3594 | (6·4) | 242 | (8·0) |  |
| History of lymphoma | 1402 | (2·52 | 43 | (1·4) |  |
| History of metastases | 3102 | (5·6) | 97 | (3·2) |  |
| History of leukaemia | 2123 | (3·8) | 78 | (2·5) |  |
| **Clinical Frailty Score** |  |  |  |  |  |
| CFS, mean (SD) | 4·0 | (1·6) | 4·0 | (1·6) |  |
| Highest temperature (˚C), mean (SD) | 37·55 | (1·0) | 37·63 | (0·98) |  |
| Highest heart rate (bpm), mean (SD) | 106 | (23) | 107 | (22) |  |
| Lowest mean arterial pressure (mmHg), mean (SD) | 63 | (11) | 63 | (10) |  |
| **Organ supportive therapies No. (%)** |  |  |  |  |  |
| Inotropes/vasopressor use | 28,729 | (51·6) | 999 | (56·5) |  |
| Invasive mechanical ventilation | 13,058 | (23·4) | 511 | (28·0) |  |
| Renal replacement therapy | 4232 | (7·6) | 103 | (6·25) |  |

Abbreviations: ICU= intensive care unit, SD = standard deviation, BMI = body mass index, APACHE = Acute Physiology and Chronic Health Evaluation, SOFA = Sequential Organ Failure Assessment, ANZROD = Risk of Death, CFS = Clinical Frailty Score, µmol/L = micromoles per litre

# S1 Fig. Sepsis survival at 3 years in women and men overall and by age group


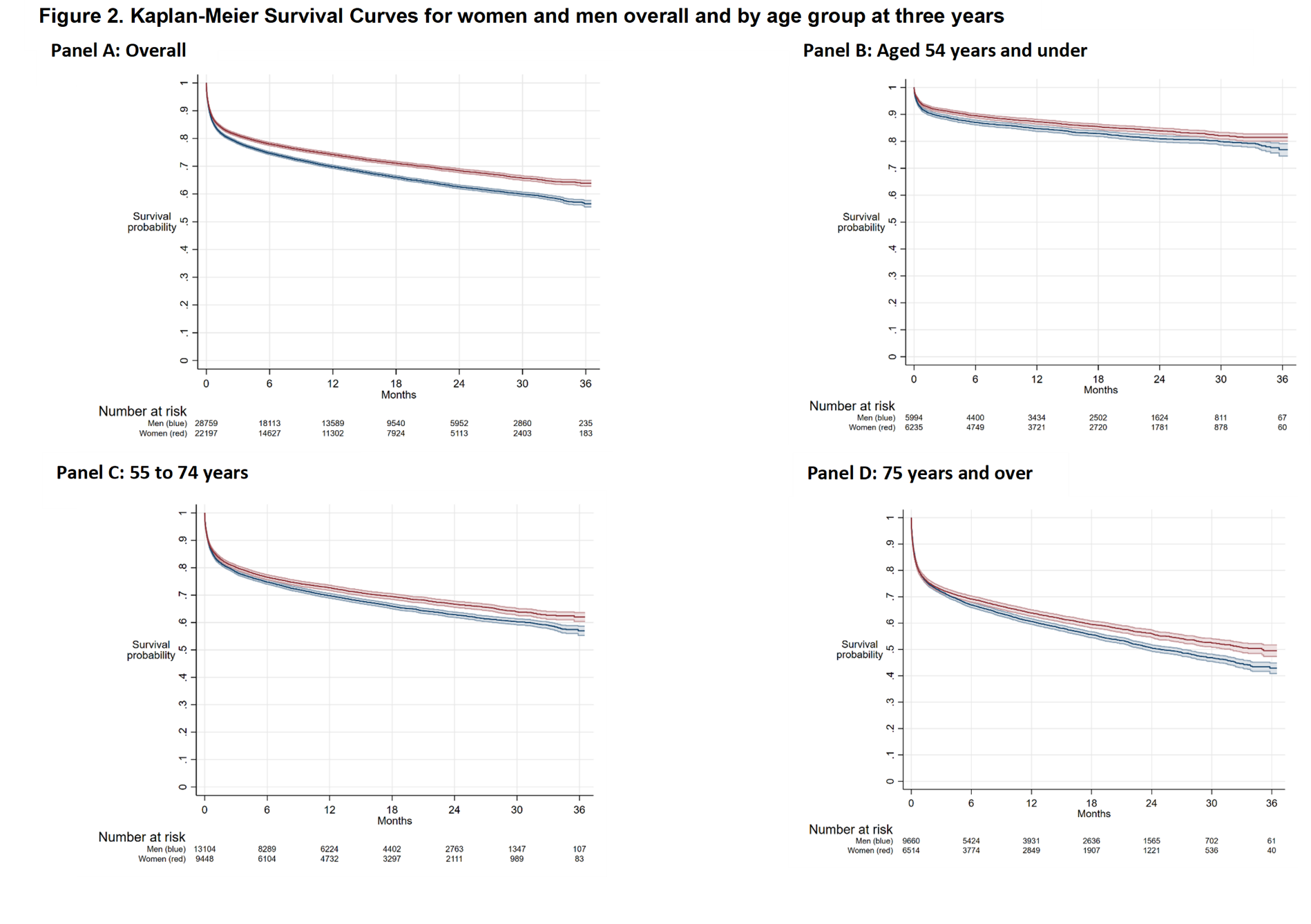


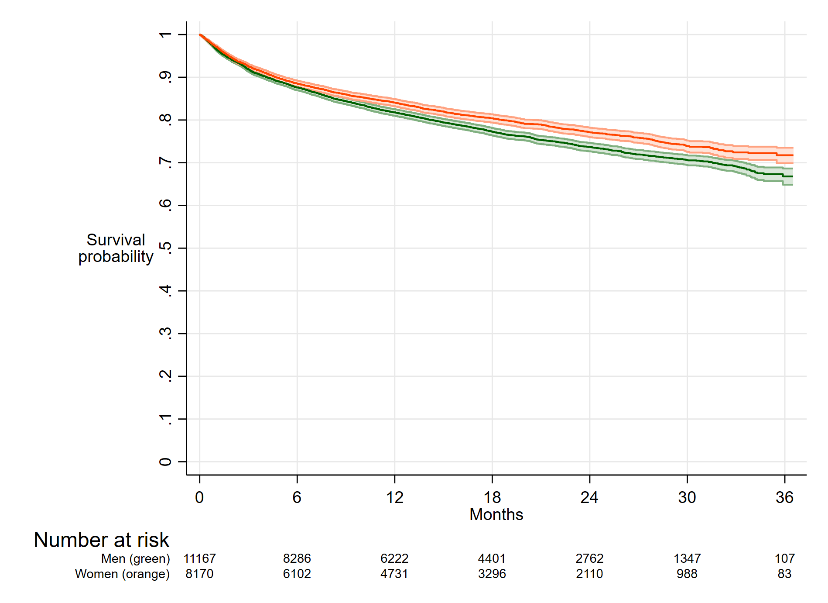

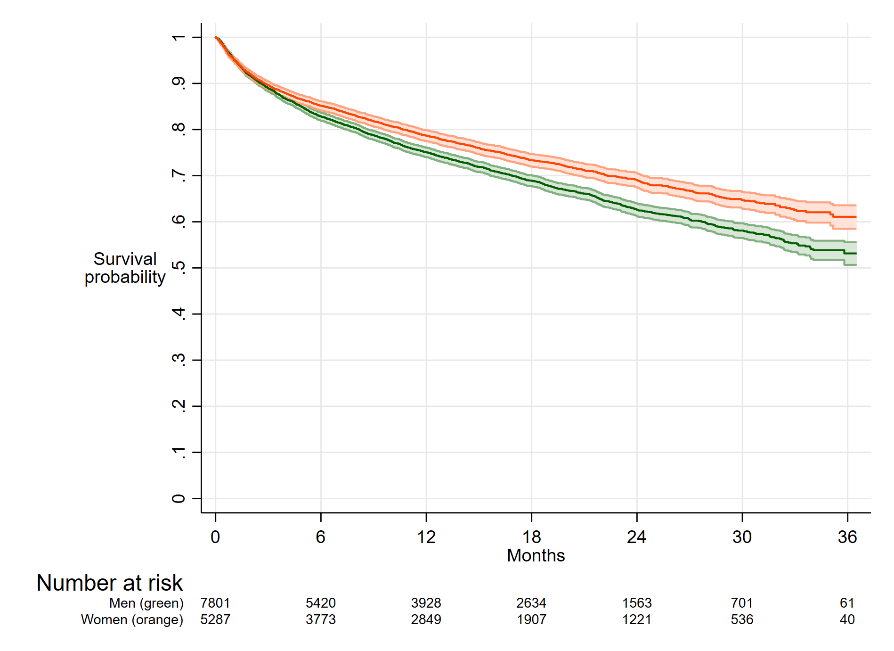

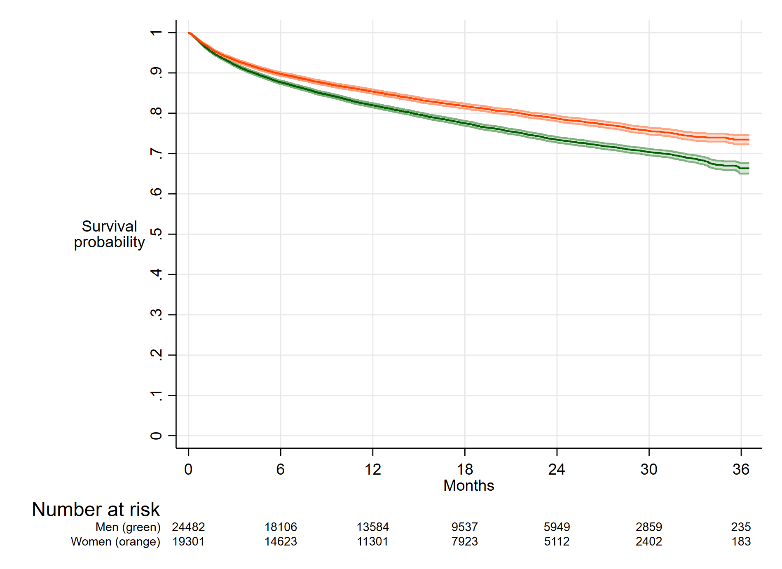

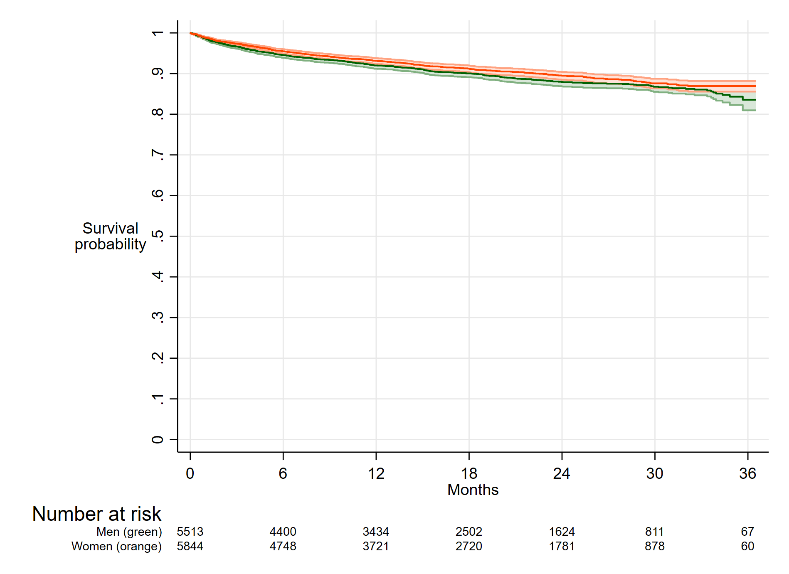


**Panel D: 75 years and over**

**Panel C: 55 to 74 years**

**Panel A: Overall**

# S2 Fig. Sepsis survival at 3 years among hospital survivors in women and men overall and by age group

**Panel B: Aged 54 years and under**

| S4 Table. Impact of individual variables on overall survival (Cox regression models for women, men and comparing men to women). | | | | | | | | | | |
| --- | --- | --- | --- | --- | --- | --- | --- | --- | --- | --- |
| **Characteristics** |  | **Women** |  |  | **Men** |  |  | **Ratio of HR men to women** | |  |
|  |  | **Hazard Ratio** | **95% CI** | | **Hazard Ratio** | **95% CI** | | **Hazard Ratio** | **95% CI** | |
| **Diagnostic group** | |  | **Lower** | **Upper** |  | **Lower** | **Upper** |  | **Lower** | **Upper** |
|  | Sepsis source unknown/not stated |  |  |  |  |  |  |  |  |  |
|  | Gastro-intestinal | 0·73 | 0·64 | 0·82 | 0·69 | 0·63 | 0·77 | 1·00 | 0·97 | 1·02 |
|  | Neurological | 0·89 | 0·73 | 1·08 | 1·05 | 0·89 | 1·24 | 1·17 | 1·14 | 1·21 |
|  | Renal/urinary/gynae | 0·64 | 0·59 | 0·69 | 0·66 | 0·62 | 0·71 | 1·04 | 1·03 | 1·05 |
|  | Respiratory | 0·93 | 0·87 | 0·99 | 1·07 | 1·02 | 1·13 | 1·16 | 1·14 | 1·17 |
|  | Skin & soft-tissue | 0·79 | 0·71 | 0·91 | 0·97 | 0·88 | 1·07 | 1·21 | 1·17 | 1·24 |
|  |  |  |  |  |  |  |  |  |  |  |
| **Age** |  | 1·023 | 1·021 | 1·025 | 1·024 | 1·022 | 1·025 | 1·00 | 1·00 | 1·00 |
| **APACHE III acute physiology score** | | 1.024 | 1·023 | 1·026 | 1·023 | 1·023 | 1·024 | 1·00 | 1·00 | 1·00 |
| **Lactate** |  |  |  |  |  |  |  |  |  |  |
|  | <2 | 1·14 | 1·06 | 1·21 | 1·10 | 1·05 | 1·16 | 0·97 | 0·96 | 0·98 |
|  | 2 to 3·9 | 1·26 | 1·14 | 1·40 | 1·21 | 1·11 | 1·31 | 0·96 | 0·94 | 0·98 |
|  | 4 to 5·9 | 1·55 | 1·36 | 1·78 | 1·43 | 1·28 | 1·60 | 0·92 | 0·90 | 0·94 |
|  | 6 to 7·9 | 2·06 | 1·74 | 2·44 | 1·96 | 1·71 | 2·25 | 0·95 | 0·92 | 0·98 |
|  | 8 to 9·9 | 2·81 | 2·31 | 3·42 | 2·50 | 2·11 | 2·95 | 0·89 | 0·91 | 0·86 |
|  | 10 to 11·9 | 3·09 | 2·48 | 3·87 | 2·29 | 1·85 | 2·83 | 0·74 | 0·75 | 0·73 |
|  | 12 to 13·9 | 3·50 | 2·95 | 4·16 | 3·31 | 2·82 | 3·89 | 0·95 | 0·95 | 0·94 |
|  | 14+ | 1·19 | 1·10 | 1·29 | 1·23 | 1·16 | 1·31 | 1·04 | 1·02 | 1·05 |
|  | Missing | 1·14 | 1·06 | 1·21 | 1·10 | 1·05 | 1·16 | 0·97 | 0·96 | 0·98 |
| **BMI** |  |  |  |  |  |  |  |  |  |  |
|  | <18·5 | 1·58 | 1·35 | 1·84 | 1·63 | 1·41 | 1·88 | 1·03 | 1·02 | 1·04 |
|  | 18·5 to 24·9 |  |  |  |  |  |  |  |  |  |
|  | 25 to 29·9 | 1·12 | 1·01 | 1·23 | 1·10 | 1·02 | 1·18 | 0·98 | 0·96 | 1·01 |
|  | 30 to 34·9 | 0·94 | 0·83 | 1·05 | 0·77 | 0·70 | 0·84 | 0·82 | 0·80 | 0·85 |
|  | 35+ | 0·85 | 0·76 | 0·95 | 0·85 | 0·77 | 0·94 | 1·00 | 0·99 | 1·02 |
|  | Unknown | 1·13 | 1·04 | 1·22 | 1·05 | 0·99 | 1·11 | 0·93 | 0·91 | 0·95 |
| **Chronic comorbidities** | |  |  |  |  |  |  |  |  |  |
|  | Respiratory | 1·23 | 1·14 | 1·34 | 1·19 | 1·12 | 1·27 | 0·97 | 0·95 | 0·98 |
|  | Cardiovascular | 1·08 | 0·99 | 1·18 | 1·05 | 0·98 | 1·12 | 0·97 | 0·95 | 0·99 |
|  | Liver (cirrhosis) | 1·94 | 1·70 | 2·22 | 1·81 | 1·63 | 2·01 | 0·93 | 0·91 | 0·96 |
|  | Renal (dialysis dependent) | 1·20 | 1·09 | 1·32 | 1·07 | 0·99 | 1·15 | 0·89 | 0·87 | 0·91 |
|  | Immunosuppressed by disease | 1·08 | 0·98 | 1·19 | 0·99 | 0·92 | 1·08 | 0·92 | 0·90 | 0·94 |
|  | Immunosuppressed by therapy | 1·33 | 1·24 | 1·44 | 1·30 | 1·22 | 1·38 | 0·98 | 0·96 | 0·99 |
|  | Lymphoma | 1·51 | 1·30 | 1·76 | 1·44 | 1·29 | 1·60 | 0·95 | 0·91 | 0·99 |
|  | Leukaemia | 1·65 | 1·46 | 1·86 | 1·69 | 1·54 | 1·84 | 1·02 | 0·99 | 1·06 |
|  | Metastatic cancer | 2·93 | 2·69 | 3·19 | 2·35 | 2·20 | 2·52 | 0·80 | 0·79 | 0·82 |
| **Clinical frailty score** | |  |  |  |  |  |  |  |  |  |
|  | Not frail (CFS < 5) |  |  |  |  |  |  |  |  |  |
|  | Pre-frail (CFS 5 or 6) | 1·57 | 1·47 | 1·69 | 1·47 | 1·39 | 1·55 | 0·93 | 0·92 | 0·94 |
|  | Frail (CFS >6) | 2·09 | 1·90 | 2·31 | 2·00 | 1·84 | 2·18 | 0·96 | 0·94 | 0·97 |
|  | Unknown | 1·38 | 1·28 | 1·47 | 1·22 | 1·16 | 1·29 | 0·89 | 0·87 | 0·90 |
| **Source of admission to hospital** | |  |  |  |  |  |  |  |  |  |
|  | Home |  |  |  |  |  |  |  |  |  |
|  | Other acute hospital (not ICU) | 1·06 | 0·98 | 1·15 | 1·11 | 1·04 | 1·18 | 1·04 | 1·03 | 1·06 |
|  | Other hospital ICU | 1·50 | 1·31 | 1·72 | 1·30 | 1·15 | 1·48 | 0·87 | 0·86 | 0·87 |
|  | Nursing home / chronic care / palliative care | 1·63 | 1·32 | 2·02 | 1·37 | 1·16 | 1·61 | 0·84 | 0·80 | 0·88 |
|  | Rehabilitation facility | 1·31 | 1·02 | 1·69 | 1·42 | 1·16 | 1·75 | 1·08 | 1·04 | 1·14 |
|  | Other (incl. mental health, inborn & unknown) | 0·97 | 0·72 | 1·29 | 0·95 | 0·77 | 1·19 | 0·99 | 0·92 | 1·06 |
| **Source of admission to ICU** | |  |  |  |  |  |  |  |  |  |
|  | Operating theatre/recovery |  |  |  |  |  |  |  |  |  |
|  | Emergency department | 1·13 | 0·98 | 1·31 | 1·34 | 1·19 | 1·50 | 1·18 | 1·15 | 1·22 |
|  | Ward | 1·59 | 1·36 | 1·86 | 1·65 | 1·45 | 1·87 | 1·04 | 1·01 | 1·07 |
|  | ICU same hospital | 1·54 | 0·72 | 3·29 | 2·04 | 1·05 | 3·97 | 1·33 | 1·21 | 1·46 |
|  | Other hospital (incl. ICU) | 1·10 | 0·92 | 1·30 | 1·31 | 1·14 | 1·50 | 1·19 | 1·15 | 1·23 |
|  | Other / unknown | 1·43 | 0·76 | 2·71 | 1·64 | 1·04 | 2·59 | 1·15 | 0·95 | 1·38 |
| **Admission following medical emergency team response** | | |  |  |  |  |  |  |  |  |
|  | No |  |  |  |  |  |  |  |  |  |
|  | Yes | 1·01 | 1·01 | 1·10 | 0·99 | 0·92 | 1·06 | 0·97 | 0·96 | 0·99 |
|  | Unknown | 1·06 | 1·06 | 1·30 | 1·28 | 1·10 | 1·49 | 1·20 | 1·15 | 1·26 |
|  |  |  |  |  |  |  |  |  |  |  |
| **Planned admission after elective surgery** | | 1.01 | 0·81 | 1·26 | 0·97 | 0·80 | 1·18 | 0·96 | 0·93 | 0·99 |
| **Ventilated on day one of ICU** | | 1.12 | 1·04 | 1·20 | 1·05 | 1·00 | 1·12 | 0·94 | 0·93 | 0·95 |
| **Treatment limitations on admission to ICU** | |  |  |  |  |  |  |  |  |  |
|  | Full active management |  |  |  |  |  |  |  |  |  |
|  | Treatment limitation | 1·87 | 1·76 | 1·99 | 1·92 | 1·82 | 2·02 | 1·02 | 1·01 | 1·03 |
|  | Treatment goal unknown / not stated | 2·11 | 1·43 | 3·13 | 1·53 | 1·03 | 2·30 | 0·73 | 0·73 | 0·72 |
| **Year of ICU admission** | |  |  |  |  |  |  |  |  |  |
|  | 2018 |  |  |  |  |  |  |  |  |  |
|  | 2019 | 0·95 | 0·95 | 0·95 | 1·04 | 0·99 | 1·09 | 1·09 | 1·08 | 1·10 |
|  | 2020 | 0·99 | 0·99 | 0·99 | 0·95 | 0·90 | 1·01 | 0·96 | 0·95 | 0·97 |
| **Hospital type** | |  |  |  |  |  |  |  |  |  |
|  | Rural/regional |  |  |  |  |  |  |  |  |  |
|  | Metropolitan | 0·95 | 0·88 | 1·03 | 1·00 | 0·94 | 1·07 | 1·05 | 1·04 | 1·07 |
|  | Tertiary | 1·04 | 0·96 | 1·12 | 1·13 | 1·06 | 1·21 | 1·09 | 1·08 | 1·11 |
|  | Private | 1·13 | 1·02 | 1·25 | 1·05 | 0·96 | 1·14 | 0·93 | 0·91 | 0·95 |
| **Region of Australia** | |  |  |  |  |  |  |  |  |  |
|  | NSW-ACT |  |  |  |  |  |  |  |  |  |
|  | QLD | 0·99 | 0·91 | 1·07 | 1·00 | 0·94 | 1·07 | 1·01 | 1·00 | 1·03 |
|  | SA-NT-WA | 0·98 | 0·91 | 1·05 | 0·99 | 0·93 | 1·05 | 1·01 | 1·00 | 1·02 |
|  | VIC-TAS | 0·99 | 0·92 | 1·05 | 1·00 | 0·95 | 1·06 | 1·02 | 1·00 | 1·03 |
| **Socio-economic status (index of relative social advantage & disadvantage)** | | | | |  |  |  |  |  |  |
|  | Lowest quintile |  |  |  |  |  |  |  |  |  |
|  | Second lowest quintile | 1·02 | 0·94 | 1·10 | 1·00 | 0·94 | 1·07 | 0·98 | 0·97 | 1·00 |
|  | Middle quintile | 0·96 | 0·89 | 1·04 | 0·97 | 0·91 | 1·03 | 1·00 | 0·99 | 1·02 |
|  | Second highest quintile | 0·96 | 0·88 | 1·04 | 0·95 | 0·89 | 1·02 | 1·00 | 0·98 | 1·02 |
|  | Highest quintile | 0·94 | 0·87 | 1·03 | 1·00 | 0·93 | 1·07 | 1·05 | 1·04 | 1·07 |
|  | Unknown (ie no postcode) | 1·11 | 0·81 | 1·51 | 1·26 | 1·01 | 1·58 | 1·14 | 1·04 | 1·25 |

Abbreviations: re. = reference, BMI = Body Mass Index, ICU= intensive care unit, SD = standard deviation, BMI = body mass index, APACHE = Acute Physiology and Chronic Health Evaluation, SOFA = Sequential Organ Failure Assessment, ANZROD = Risk of Death, CFS = Clinical Frailty Score. NSW = New South Wales, ACT = Australian Capital Territory, QLD = Queensland, SA = South Australia, NT = Northern Territory, WA = Western Australia, VIC = Victoria, TAS = Tasmania

# S3 Fig. COVID patients only.


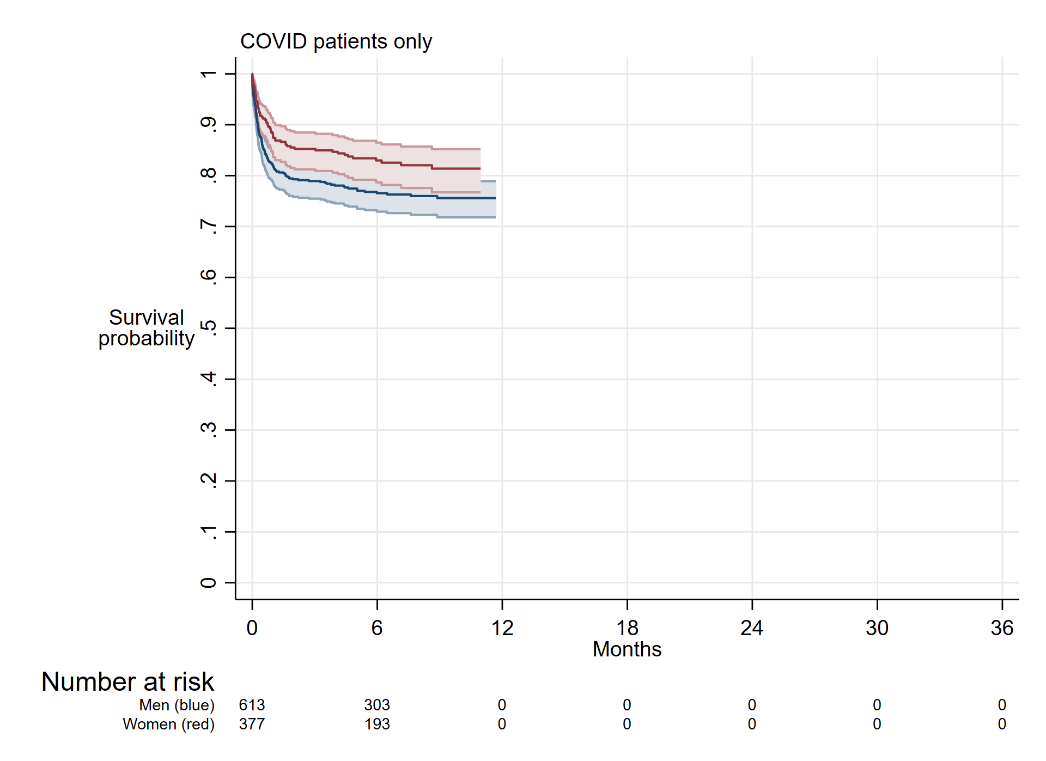


Survival at 3 years among women and men with SARS-CoV-2.

# S4 Fig. COVID patients excluded.


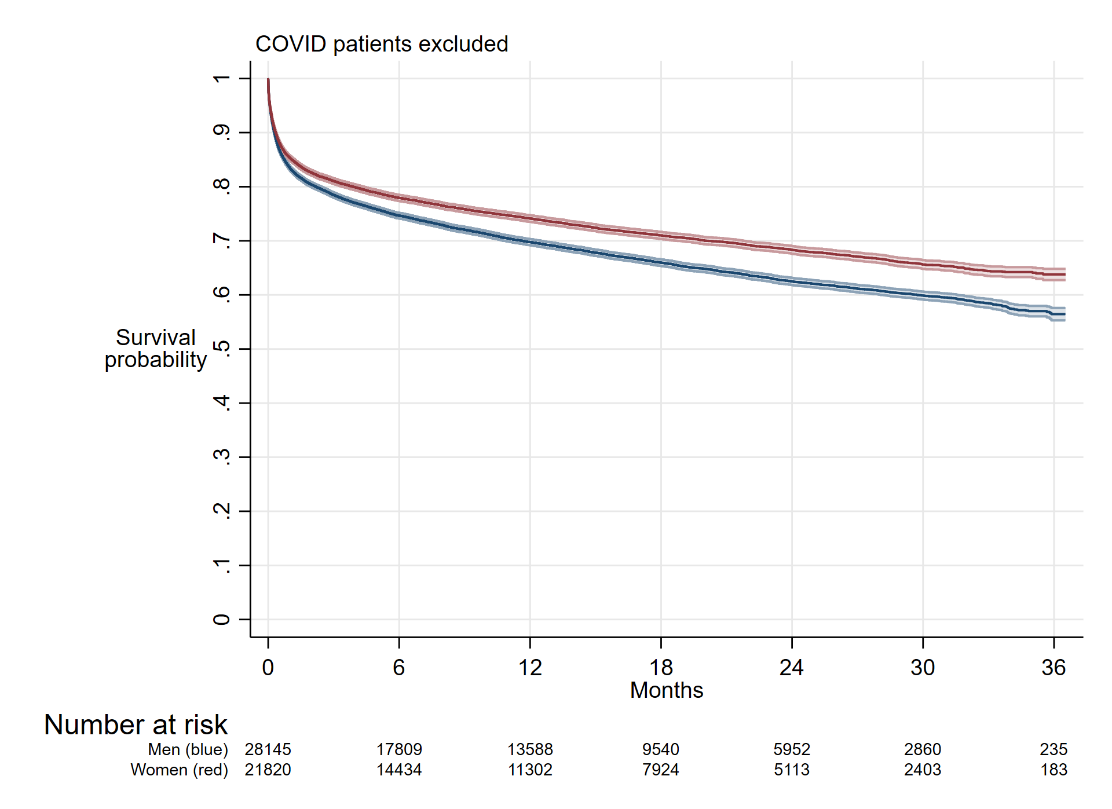


Sepsis survival excluding SARS-CoV-2 patients in women and men overall.
