# Supplementary material for: Sex differences in long-term survival after intensive care unit treatment for sepsis: A cohort study
Source: PLoS One. 2023 Feb 24;18(2):e0281939. doi: 10.1371/journal.pone.0281939 (PMC9955961; doi:10.1371/journal.pone.0281939)
Supplement: Attachment — Submitted filename: Response to reviewers_28Jan23.doc [file pone.0281939.s002.doc]

24 January 2023

Editor-in-Chief

PLOS ONE

Dear Editor-in-Chief

**RE: PONE-D-22-30745: Sex differences in survival after intensive care unit treatment for sepsis: a cohort study**

# Thank you PLOS ONE Editors and Reviewers for your thoughtful review of our manuscript. Please find below our response to reviewer and academic editor feedback which we believe has considerably improved the quality of our manuscript.

1. Please ensure that your manuscript meets PLOS ONE's style requirements, including those for file naming.

*Response: The files have been updated in accordance with PLOS ONE’s style requirements.*

2. We noticed you have some minor occurrence of overlapping text with the following previous publication(s), which needs to be addressed:

-https://doi.org/10.1016/j.jinf.2022.04.035
-10.1001/jamaneurol.2018.0123

In your revision ensure you cite all your sources (including your own works), and quote or rephrase any duplicated text outside the methods section. Further consideration is dependent on these concerns being addressed.

*Response: As the corresponding author of the first of these papers, I have compared the manuscripts in Microsoft word and cannot see any significant overlapping text which would raise plagiarism concerns. My colleague David Pilcher, the senior author on this paper has done the same for the JAMA Neurology paper and has also found minimal overlapping text outside of the methods section. Please note, the description of the ANZICS APD in the methodology section is similar in all papers that are written using this bi-national registry. It is written in this way to comply with ANZICS requirements and is specifically to ensure consistency of description throughout all publications.*

3. Thank you for stating the following financial disclosure:

"KT received funding for the work from the National Health and Medical Research Council of Australia"
 
Please state what role the funders took in the study.  If the funders had no role, please state: "The funders had no role in study design, data collection and analysis, decision to publish, or preparation of the manuscript." If this statement is not correct you must amend it as needed.

Please include this amended Role of Funder statement in your cover letter; we will change the online submission form on your behalf.

*Response: Thank you for updating the online submission form on our behalf. The manuscript funding section at the end of the paper has been updated in tracked changes accordingly to confirm that: "The funders had no role in study design, data collection and analysis, decision to publish, or preparation of the manuscript."*

4. We note that you have indicated that data from this study are available upon request. PLOS only allows data to be available upon request if there are legal or ethical restrictions on sharing data publicly. For more information on unacceptable data access restrictions, please see [http://journals.plos.org/plosone/s/data-availability#loc-unacceptable-data-access-restrictions](https://nam12.safelinks.protection.outlook.com/?url=http%3A%2F%2Fjournals.plos.org%2Fplosone%2Fs%2Fdata-availability%23loc-unacceptable-data-access-restrictions&data=05|01||6e38f0dff22c47ba7c4808dafa03c4bd|84df9e7fe9f640afb435aaaaaaaaaaaa|1|0|638097191384173488|Unknown|TWFpbGZsb3d8eyJWIjoiMC4wLjAwMDAiLCJQIjoiV2luMzIiLCJBTiI6Ik1haWwiLCJXVCI6Mn0%3D|3000|||&sdata=pF%2FzObavg7voab%2B73eKkFDSz9MhcH9Rpv8HzKYnMkXc%3D&reserved=0).

In your revised cover letter, please address the following prompts:

a) If there are ethical or legal restrictions on sharing a de-identified data set, please explain them in detail (e.g., data contain potentially sensitive information, data are owned by a third-party organization, etc.) and who has imposed them (e.g., an ethics committee). Please also provide contact information for a data access committee, ethics committee, or other institutional body to which data requests may be sent.

b) If there are no restrictions, please upload the minimal anonymized data set necessary to replicate your study findings as either Supporting Information files or to a stable, public repository and provide us with the relevant URLs, DOIs, or accession numbers. For a list of acceptable repositories, please see [http://journals.plos.org/plosone/s/data-availability#loc-recommended-repositories](https://nam12.safelinks.protection.outlook.com/?url=http%3A%2F%2Fjournals.plos.org%2Fplosone%2Fs%2Fdata-availability%23loc-recommended-repositories&data=05|01||6e38f0dff22c47ba7c4808dafa03c4bd|84df9e7fe9f640afb435aaaaaaaaaaaa|1|0|638097191384173488|Unknown|TWFpbGZsb3d8eyJWIjoiMC4wLjAwMDAiLCJQIjoiV2luMzIiLCJBTiI6Ik1haWwiLCJXVCI6Mn0%3D|3000|||&sdata=WheaX61ApTidsLv08RbQ6kr%2FghIqAdXUQqoQm6GL9No%3D&reserved=0).

We will update your Data Availability statement on your behalf to reflect the information you provide.

*Response: Thank you for updating this on our behalf. The data availability statement should be as follows:*

*“Deidentified individual patient data are collected from each hospital ICU under Quality Assurance Legislation and standing agreements with each State and Territory health department of Australia and the Ministry of Health of New Zealand. Data are collected primarily for the purpose of benchmarking ICU activity and performance. Secondary access and use of the data is restricted other than under specific agreements with researchers. Applications for access to data can be made to* [*anzics.core@anzics.com.au*](mailto:anzics.core@anzics.com.au)*”*

5. We note that you have stated that you will provide repository information for your data at acceptance. Should your manuscript be accepted for publication, we will hold it until you provide the relevant accession numbers or DOIs necessary to access your data. If you wish to make changes to your Data Availability statement, please describe these changes in your cover letter and we will update your Data Availability statement to reflect the information you provide.

*Response: Please see the above response including the updated data availability statement.*

6. Please upload a new copy of Figure 2 and 3 as the detail is not clear. Please follow the link for more information:

*Response: A new copy of Figure 2 has been uploaded. Please note, Figure 3 has now been separated into 2 figures, (Figs 3 and 4) to improve readability and clarity of text in the figure.*

7. Please include captions for your Supporting Information files at the end of your manuscript, and update any in-text citations to match accordingly. Please see our Supporting Information guidelines for more information: <http://journals.plos.org/plosone/s/supporting-information>.

*Response: A new section with Supporting information file captions has been included at the end of the manuscript. Please confirm whether this is appropriate? After reading the supporting information instructions I was unsure as to whether the journal requires the figures and tables in the Supporting Information file to be uploaded to the manuscript.*

8. Please review your reference list to ensure that it is complete and correct. If you have cited papers that have been retracted, please include the rationale for doing so in the manuscript text, or remove these references and replace them with relevant current references. Any changes to the reference list should be mentioned in the rebuttal letter that accompanies your revised manuscript. If you need to cite a retracted article, indicate the article’s retracted status in the References list and also include a citation and full reference for the retraction notice.

*Response: No changes to the reference list were made.*

**Comments to the Author**

Reviewer #1: 

Comments:

Fig 3: what is the ref for CFS? OR is worse for CFS 5-6 than for CSF 7 an-8? Please comment

*Response: The reference group for the Clinical Frailty Score is ‘not frail’(the top HR reported). The most frail group (CFS >6) had the highest HR in both women and men. The HR at the bottom is for those with an unknown frailty score. Please let us know if further clarification is required?*

The inclusion period was partly during the COVID-19 surge period. Did you analyse specifically the impact of sex in this population? See for exemple (Sex-specific treatment characteristics and 30-day mortality outcomes of critically ill COVID-19 patients over 70 years of age-results from the prospective COVIP study; Canadian Journal of Anaesthesia. 2022-08-09)

*Response: Thank you for this comment. We included patients with COVID but did not examine COVID as a subgroup as this group represented a small component of the whole study population due to relatively few COVID admissions to ICU in Australia in 2020.*

*On re-examining the data, there were 990 probable COVID positive patients included (which includes some probable COVID positive patients from right at the beginning of the pandemic who didn’t get definitive tests on admission but were likely positive). Of these 337 were female (9.8% mortality) and 613 male (16.3% mortality). There were 456 confirmed COVID positive pneumonitis patients. Of these 163 were female (11.0% mortality) and 293 were male (15.7% mortality). We have now made a comment on this in the main manuscript and have reported additional information related to SARS-CoV-2 survival for females and males in the Supporting Information (Figs S3 and S4).*

Reviewer #2: 
The authors postulated that greater protection in women may be attributed to the immune enhancing effects of estrogen, and male disadvantage may be due to the immunosuppressive properties of male androgens, particularly testosterone. In this regard, testosterone is common intermediate between males and females and it gets converted enzymatically to 17β-estradiol by Aromatase or to 5α-dihydrotestosterone (DHT) by 5α-reductase. Thus, DHT rather than testosterone might be the active immunosuppressive steroid.

Additionally, estrogen decrease with aging in women and clearly the majority of the women in this study were post-menopausal with low estrogen levels. It is therefore possible that post-menopausal women in this study were on HRT and thus they had better survival than men. However, hormonal status nor information about HRT was not provided in this study. In men, the levels of DHT also decrease with aging and thus aged men should do better than women – this, however, was not the case. So, the reason for the difference between men and women in survival remains unclear. Could it be due to a difference in the estrogen to DHT ratio? Unfortunately, this information was also not available in this study. Furthermore, appropriate explanation/justification for the difference in present from those of Lin S et al 2021 need to be provided.

*Response: As the reviewer points out, we did not have any data related to women’s post-menopausal status (beyond age) or their use of Hormone Replacement Therapy. We agree that it is possible that 5α-dihydrotestosterone (DHT) may have had a role in male immunosuppression and have added this in the discussion section.*

*In regards to the different findings in our study compared to the study conducted by Lin et al in Boston, one possibility could be the difference in follow-up time. We have added this in tracked changes to our discussion section.*

Thank you for your reconsideration of the manuscript for publication in PLOS ONE.

Kind Regards

**Dr Kelly Thompson**

Conjoint Senior Lecturer, UNSW

Research Fellow, The George Institute for Global Health
